# Supplementary material for: Problem-Based Learning Discussion to Introduce Quality Improvement to Residents in the Perioperative Setting
Source: MedEdPORTAL. 2021 Nov 29;17:11198. doi: 10.15766/mep_2374-8265.11198 (PMC8627916; doi:10.15766/mep_2374-8265.11198)
Supplement: Supplementary file 1 — Staff Feedback Questionnaire.docxPre-PBLD Learner Survey.docxCase Stem and Required Reading.docxPost-PBLD Learner Survey.docxModel Learning Discussion.docx [file mep_2374-8265.11198-s001.zip › B. Pre-PBLD Learner Survey.docx]

**Pre-PBLD Learner Opinions on QI Survey**

1. I think learning about Quality Improvement is an important part of learning to be an anesthesiologist.
   1. Strongly Disagree
   2. Disagree
   3. Neutral
   4. Agree
   5. Strongly Agree
2. Learning about Quality Improvement will prepare me for success on in training and board examinations.
   1. Strongly Disagree
   2. Disagree
   3. Neutral
   4. Agree
   5. Strongly Agree
3. I will use Quality Improvement in my day to day practice as an anesthesiologist.
   1. Strongly Disagree
   2. Disagree
   3. Neutral
   4. Agree
   5. Strongly Agree
4. The only reason I will complete a Quality Improvement project is because the Accreditation Council on Graduate Medical Education has made it a requirement.
   1. Strongly Disagree
   2. Disagree
   3. Neutral
   4. Agree
   5. Strongly Agree
5. I am confident that I can complete a Quality Improvement project that will positively impact others
   1. Strongly Disagree
   2. Disagree
   3. Neutral
   4. Agree
   5. Strongly Agree
