# Supplementary material for: Problem-Based Learning Discussion to Introduce Quality Improvement to Residents in the Perioperative Setting
Source: MedEdPORTAL. 2021 Nov 29;17:11198. doi: 10.15766/mep_2374-8265.11198 (PMC8627916; doi:10.15766/mep_2374-8265.11198)
Supplement: Supplementary file 1 — Staff Feedback Questionnaire.docxPre-PBLD Learner Survey.docxCase Stem and Required Reading.docxPost-PBLD Learner Survey.docxModel Learning Discussion.docx [file mep_2374-8265.11198-s001.zip › C. Case Stem and Required Reading.docx]

**Problem Based Learning Discussion** (PBLD)
**Quality Improvement in the Perioperative Space**

**Case Stem and Learning Discussion**

By the end of this activity, learners will be able to:

1. State the purpose and value of Improvement in the healthcare setting.
2. Describe the differences between Patient Safety, Quality Improvement, and Quality Assurance in the healthcare setting.
3. Describe the risks and responsibilities common in Improvement.
4. List the phases of a Quality Improvement project.
5. Apply Quality Improvement concepts to a perioperative scenario.

Case
After graduating from your anesthesiology residency, you join a medium sized group practice as a board eligible anesthesiologist. This practice operates primarily at a 300-bed suburban hospital. As the newest member of the group, you are given the job of addressing hospital administration concerns about the number of patients that stay in the post-anesthesia care unit (PACU) for longer than 3 hours. The department’s chief reminds you that long PACU stays require operating room (OR) teams to wait until a recovery bed is available before beginning the next surgical case and PACU personnel to stay until all patients have sufficiently recovered. This has led to an increase in overtime costs and decreased morale. She says, “You must have done some Quality Improvement (QI) in your residency. You have more experience than anybody else in our department to solve this problem.” The QI project you worked on as a resident was already started by the time you arrived and you are unsure where to start with a brand-new project. You are eager to do what you can to improve the care of your patients, but you always pictured that meant saving the day with a dramatic intubation or a diagnosis everyone else missed. You think, “Do attending anesthesiologists still need to improve? I thought they would have figured it out by now.”

Discussion:

1. What is the purpose of Quality Improvement in medical practice?
2. What is unique about the role of an anesthesiologist that facilitates improvement activities? What would you like to improve about your patients’ care?

As you look more into the purpose of QI this task feels more and more important to get correct. You wonder if everyone will listen to the solutions you come up with. You are the new person, how are you going to understand the way the system works? It seems like Improvement has come up more lately compared to when you were in medical school. Why is this so important all the sudden?

Discussion:

1. Can you solve this problem yourself? Do you need to include anyone else?
2. Why do specialty professional organizations like the American Board of Anesthesiology care that anesthesiologists know how to perform Improvement?

It seems like the personnel in the Patient Safety division of your hospital talk an awful lot about QI projects. Is QI the same thing as Patient Safety? You know that you are supposed to work with your department quality officer to report Quality Assurance (QA) outcomes. Is QI the same thing as QA?

Discussion:

1. Define the differences between Patient Safety, Quality Improvement, and Quality Assurance.

You talk about the differentiation of Improvement and Patient Safety with your department head to find out exactly what it is she wants you to do. “That all sounds like research to me. I don’t want to get in trouble. Make sure you talk to the IRB before you do anything,” she says.

Discussion:

1. Differentiate QI from human subjects research.
2. What responsibilities to patients do physicians have when practicing Improvement?

You have created a team of stakeholders interested in decreasing the number of patients with prolonged PACU stays consisting of other anesthesia providers, nurses from the PACU, and a hospital level administrator. None of the members of the team have completed a QI project but they all assure you that they are eager to help in any way they can. You wonder, “Where do I even begin?”

Discussion:

1. What are the stages of a QI project?
   The department chief wants an update on your progress. Given the complexity of your system you are struggling with how to present you progress in a concise manner.

Discussion:

1. How can an A3 template help to communicate your project’s progress?

Now that we have outlined the purpose and process of QI for anesthesiologists, let’s apply some of these concepts and tools. We will work through our PACU problem.

Discussion:

1. What do you want to know about the system you are improving?

Investigation of long PACU stays providers the following information over the course of the month:

| Number of PACU recoveries | Average PACU Stay (hrs) | Number of stays over 3 hours | Proportion of PACU Stays over 3 hours | Proportion of prolonged stay for pain | Proportion of prolonged stay for nausea | Proportion of prolonged stay for sedation |
| --- | --- | --- | --- | --- | --- | --- |
| 1156 | 2.1 | 347 | 0.3 | 0.05 | 0.69 | 0.26 |

There does not appear to be a pattern to the prolonged stays in terms of the type of surgical procedure or the involved personnel. Based on this info you feel that a large proportion of patients who have a prolonged PACU stay are due to postoperative nausea and vomiting (PONV).

Discussion:

1. Develop a concise the goal for improvement, describe the root cause of the current state, and define the target condition for improvement.

Further analysis into PONV in this group yielded the following information:

| Number of PACU recoveries > 3 hrs | Proportion of patients receiving inhalational anesthesia | Proportion of patients receiving one antiemetic in OR | Proportion of patients receiving two antiemetics in OR | Cost in antiemetic(s) per patient |
| --- | --- | --- | --- | --- |
| 347 | 0.98 | 0.72 | 0.23 | $8.96 |

Based on this analysis your team decides to add pre and postoperative PONV order sets with selectable medications for prophylaxis and treatment to the electronic health record.

Discussion:

1. Develop an intervention. Outline the implementation and follow up for this intervention.

Your team gathered the following results for the 2-week time period for the pilot group:

| Proportion of PACU stays > 3 hours | Proportion of patients receiving one antiemetic | Proportion of patients receiving two antiemetics | Cost in antiemetic(s) per patient |
| --- | --- | --- | --- |
| 0.19 | 1.0 | 1.0 | $111.29 |

Just then you get a call from the department chief for what you assume will be a thank you for decreasing the proportion of PACU stays longer than 3 hours. Instead, she is very irate. Pharmacy is breathing down her neck because the cost of antiemetics used by the department in the last 2 weeks has spiked. Anesthesia providers seem to be premedicating more patients with aprepitant (which was included in your order sets) lately.

Discussion:

1. Is a QI project that does not achieve the desired end state a failure? Once a pilot group has encountered an unexpected problem, what is the next step?

Required reading prior to problem based learning discussion:

Fleisher LA. Quality Anesthesia: Medicine Measures, Patients Decide. Anesthesiology. 2018;129:1063–1069. <https://doi.org/10.1097/ALN.0000000000002455>

Silver SA, Harel Z, McQuillan R, et al. *Clinical Journal of the American Society of Nephrology.* 2016; 11(5): 893-900. <https://doi.org/10.2215/CJN.11491015>

Bassuk JA, Washington IM. The a3 problem solving report: a 10-step scientific method to execute performance improvements in an academic research vivarium. *PLoS One*. 2013;8(10):e76833. Published 2013 Oct 29. <https://doi.org/10.1371/journal.pone.0076833>
