# Supplementary material for: Problem-Based Learning Discussion to Introduce Quality Improvement to Residents in the Perioperative Setting
Source: MedEdPORTAL. 2021 Nov 29;17:11198. doi: 10.15766/mep_2374-8265.11198 (PMC8627916; doi:10.15766/mep_2374-8265.11198)
Supplement: Supplementary file 1 — Staff Feedback Questionnaire.docxPre-PBLD Learner Survey.docxCase Stem and Required Reading.docxPost-PBLD Learner Survey.docxModel Learning Discussion.docx [file mep_2374-8265.11198-s001.zip › D. Post-PBLD Learner Survey.docx]

**QI Simulation Feedback Survey**

Based on feedback survey created in: Greenlaw C, Jacob S, Cheston C. Pediatric quality improvement (QI) virtual practicum: adapting a QI simulator. MedEdPORTAL. 2020; 16: 10929. https://doi.org/10.15766/mep_2374-8265.10929

|  | Strongly Disagree | Disagree | Neutral | Agree | Strongly Agree |
| --- | --- | --- | --- | --- | --- |
| Before the PBLD, I felt completely confident in my abilities to lead a QI initiative. | 1 | 2 | 3 | 4 | 5 |
| I understood the subject matter of the scenario well enough to actively participate. | 1 | 2 | 3 | 4 | 5 |
| The PBLD provided me with a realistic scenario with a reasonable progression. | 1 | 2 | 3 | 4 | 5 |
| I gained valuable experience in completing this PBLD. | 1 | 2 | 3 | 4 | 5 |
| After the PBLD, I feel more confident in my abilities to lead a QI initiative. | 1 | 2 | 3 | 4 | 5 |
| After the PBLD, I am more likely to start my own QI initiative. | 1 | 2 | 3 | 4 | 5 |

Any additional feedback or reflections on the QI Simulation activity? Is this a good way to teach and discuss QI? What should be changed or modified for the next group of learners?
