# Supplementary material for: Problem-Based Learning Discussion to Introduce Quality Improvement to Residents in the Perioperative Setting
Source: MedEdPORTAL. 2021 Nov 29;17:11198. doi: 10.15766/mep_2374-8265.11198 (PMC8627916; doi:10.15766/mep_2374-8265.11198)
Supplement: Supplementary file 1 — Staff Feedback Questionnaire.docxPre-PBLD Learner Survey.docxCase Stem and Required Reading.docxPost-PBLD Learner Survey.docxModel Learning Discussion.docx [file mep_2374-8265.11198-s001.zip › E. Model Learning Discussion.docx]

**Problem Based Learning Discussion**
**Quality Improvement in the Perioperative Setting**

**Model Learning Discussion**
After graduating from your anesthesiology residency, you join a medium sized group practice as a board eligible anesthesiologist. This practice operates primarily at a 300-bed suburban hospital. As the newest member of the group, you are given the job of addressing hospital administration concerns about the number of patients that stay in the post-anesthesia care unit (PACU) for longer than 3 hours. The department’s chief reminds you that long PACU stays require operating room (OR) teams to wait until a recovery bed is available before beginning the next surgical case and PACU personnel to stay until all patients have sufficiently recovered. This has led to an increase in overtime costs and decreased morale. She says, “You must have done some Quality Improvement (QI) in your residency. You have more experience than anybody else in our department to solve this problem.” The QI project you worked on as a resident was already started by the time you arrived and you are unsure where to start with a brand-new project. You are eager to do what you can to improve the care of your patients, but you always pictured that meant saving the day with a dramatic intubation or a diagnosis everyone else missed. You think, “Do attending anesthesiologists still need to improve? I thought they would have figured it out by now.”

1. What is the purpose of Quality Improvement in medical practice?

Healthcare is expensive and dangerous. This is due in part to errors and omissions that increase morbidity and inefficiency.^1,2^ Scientific research continuously discovers new ways to provide superior healthcare while reducing costs. However, application of these advances lags well behind their discovery.^1^ Physicians typically view their practice as tailoring a specific course of treatment for each individual patient.^3,4^ Unfortunately, this is not a realistic and practical picture of our healthcare system. Pursuing this type of unstandardized approach to medical care leads to incomplete and improper application of effective medical treatment.^4^

W.E. Deming, considered the father of modern Improvement, established a process of standardization for industrial production in the 20^th^ century to maximize efficiency and quality in manufacturing.^5^ The process has since been adapted to formalize systematic Improvement in medicine. In both manufacturing and medicine standardization of processes has led to cheaper and higher quality results.^6-9^

Research shows that implementation of Improvement concepts to our healthcare systems results in better patient care outcomes.^1,4^ We are ethically obligated to provide our patients with the highest quality and most appropriate medical care at all times.^1,3^ Part of providing that care is determining what care is best for the population of patients in our particular system of care. How to best apply care is not always obvious, and the forces that impact care may change over time. Continued growth and evaluation are needed to improve and maintain a satisfactory quality of healthcare.^1^

1. What is unique about the role of an anesthesiologist that facilitates improvement activities? What would you like to improve about your patients’ care?

Anesthesia has long been recognized as a field that emphasizes Improvement.^2^ The specialty was born out of a need for improved patient safety and outcomes in the perioperative period. Because of these efforts, operative mortality has continually decreased throughout history.^2,7^ Anesthesiologists work with many surgical and non-surgical disciplines in numerous practice settings including the operating room, the intensive care unit, labor and delivery, and even outpatient procedure clinics. This allows valuable insight into the systemic causes of outcomes in these environments.^10^ More recently anesthesiologists have increasingly become more aware and engaged in safety culture, surgical checklists, and care bundles.^11^ The familiarity with these concepts make anesthesiologists well suited to engage in QI.

Anesthesiologists have profound impact on patient outcomes in the perioperative period regardless of the surgical procedure, making them well suited to address perioperative outcomes.^7^ While they may not always be the immediate cause of a perioperative event, once that event occurs, they provide care that directs the ultimate outcome. Action and leadership from the anesthesiologist often prevent the clinical environment from developing into a scenario that results in an adverse event in the first place. Anesthesiologists taking responsibility for their patient’s outcomes, regardless of the cause, improves patient care.^7^ Taking consistent ownership for patient outcomes and continuous engagement in Improvement is critical in the development of a senior physician.

The role of physicians in the healthcare team and in healthcare organizations is one of a leader. Without their leadership systematic change and therefore improvement cannot take place.^12,13^ In practice, QI can be perceived negatively by physicians because of other demands for their time and concerns that it limits the autonomy needed to practice medicine. As a result, physicians may not seek the tools and experience to engage in quality improvement. To address physicians’ concerns large organizations often use QI advisors to develop and maintain a culture of improvement.^12-15^ Physicians who are well educated in QI terminology and methodology can bridge some of these gaps and act as leaders for QI. They can be a powerful example to trainees and advocates that steer institutional care toward continued process improvement.

Once learners discuss why physicians and anesthesiologists are key to Improvement in the perioperative space, they should take a few minutes to share ideas about what needs improvement in their workspace. This will allow them to grasp the importance of leadership and engagement by physicians for improvement to occur. Those who work within the system that is being improved have the most stake in its improvement and are able to best identify a solution that benefits themselves and the work they do.^12,16^ There is a vast range of issues that learners can discuss in order to begin the exercise of recognizing the need for Improvement. Common deficiencies in medicine include areas of unscientific care, inappropriate care, high cost of care, delays in care, geographic or specialty base variations in practice, latent disagreements between clinicians, poor documentation, and unrecognized adverse patient outcomes.^11,17^ Learners can also consider how their ideas relate to the six domains of quality when generating these ideas for improvement: effective, equitable, timely, efficient, safe, and patient-centered.^18^

As you look more into the purpose of QI this task feels more and more important to get correct. You wonder if everyone will listen to the solutions you come up with. You are the new person, how are you going to understand the way the system works? It seems like Improvement has come up more lately compared to when you were in medical school. Why is this so important all the sudden?

1. Can you solve this problem yourself? Do you need to include anyone else?

It is unlikely that a meaningful and lasting improvement can be accomplished by one person providing mandates to other people on how to improve. Systems are generally too complex and require input from all of those performing the work as well as system administrators who are observing the system outcomes. A QI team should consist of a multidisciplinary group of stakeholders, each with control over a different aspect of patient care. This team can include physicians from multiple specialties, other health professionals (Pharmacists, therapists), nursing, administrators, and even patients. This diversity of expertise and perspective can expand the impact of any improvement and can facilitate the sustainment of interventions and improvements. ^19,20^ It is also important to consider the role of all team members to the QI project. Some may be highly motivated to solve a problem and others not. Likewise, some may have more power to effect change and other less. Considering where your team members fall on a grid of level of interest and power to effect systemic change can be critical to addressing the specific tasks over the course of a QI project.^12^

1. Why do specialty professional organizations like the American Board of Anesthesiology care that anesthesiologists know how to perform Improvement?

Healthcare is incredibly complex, which allows endless opportunities for improvement. Disparities in healthcare are clear and urgent examples of the need for improvement. Expansion of healthcare systems to reach groups that are underserved requires constant improvement to impact the greatest number of people in the most effective way.^12,21-23^ Organizations entrusted to foster the best care possible, for the most possible people, in the fairest possible way recognize the need for Improvement activities to reach these goals. Society expects anesthesiologists to be advocates for patients. In order to mobilize improvements in urgent times (i.e. during a global pandemic), it is necessary that these processes and teams are already in existence and capable of impacting the systems of care. Our professional organizations address this directly. The American Society of Anesthesiologists created the Anesthesia Quality Institute and the National Anesthesia Clinical Outcomes Registry to allow for institutional and cross institutional evaluation and feedback^24-27^ and the American Board of Anesthesiology has made Improvement and simulation a requirement for maintenance of certification.^28,29^ The Accreditation Council on Graduate Medical Education has also made requirements to ensure resident physicians are introduced and engaged in Improvement.^30^

Medical School curriculum generally focuses on medical knowledge, leaving teaching of improvement skills to later periods of training and practice.^31,32^ Practically speaking, residency is the first time that physicians are affected by and can impact the system of patient care. This is why the optimal time to teach physicians the principles and skills of quality improvement at the beginning of their career during residency. Residents who are already improving their practice daily may be more open to introducing quality improvement into their practice than those later in their careers. Residents also spend the most time in patient care and witness the daily impact of the care they deliver. This positions them to identify issues and implement changes that can improve patient outcomes. Introduction of quality improvement into a resident’s practice sets the framework that benefits an entire career’s worth of patients.^31,32^

It seems like the personnel in the Patient Safety division of your hospital talk an awful lot about QI projects. Is QI the same thing as Patient Safety? You know that you are supposed to work with your department quality officer to report Quality Assurance (QA) outcomes. Is QI the same thing as QA?

1. Define the differences between Patient Safety, Quality Improvement, and Quality Assurance.

Patient safety is a philosophy of addressing the management of a healthcare system. The delivery of healthcare is a very complex process which is impacted by innumerable processes. Since there are a practically infinite number of events that lead to a given patient outcome, consequently there is also an infinite number of ways to improve patient safety. By making these changes the system of healthcare delivery can be made infinitely safer by adding more and more safeguards. These safeguards all have a cost. Patient Safety does not traditionally consider these costs to the system in the same way that it addresses the likelihood of a given event occurring. In fact, the system can be made so safe that patient care is impeded, which is paradoxically unsafe for the patient.^11^

Quality Improvement addresses more than simply the likelihood of an adverse patient outcome. QI also considers if the care is effective, equitable, timely, efficient, safe, and patient-centered.^19^ QI requires an understanding of how to generate positive outcomes rather than how to avoid negative outcomes. To perform QI, physicians must understand the system, the range of possible outcomes, and how changes to the system will lead to changes in the ultimate outcome.^23,33^ QI is typically done in a group space with a multidisciplinary team working towards a common goal of improving the system in a measurable way. Statistical processes are more critical to QI than Patient Safety because demonstrating improvement generally requires analysis of observed outcomes in the setting of a given intervention. Run graphs or control charts are commonly used to track the improvement of the system across time. When interventions are made it is critical for QI that the changes to the system are analyzed so that further changes can be made and lead to further improvement. In this way the scientific method is leveraged to create the best possible system.^34^

QA also considers outcomes over time. QA tracks both outcomes and processes as a way of providing evidence of the value of care already provided.^35-38^ If reliably tracked in an established system or institution, indicators of quality are largely stable over time. This “statistical control” allows for the continuous evaluation of care and the prediction of required resources and cost.^39,40^ This is why QA is valuable to payers and healthcare societies who typically champion these types of efforts.

You talk about the differentiation of Improvement and Patient Safety with your department head to find out exactly what it is she wants you to do. “That all sounds like research to me. I don’t want to get in trouble. Make sure you talk to the IRB before you do anything,” she says.

1. Differentiate QI from human subjects research.

Improvement activities can share some similarities with human subjects research (HSR), but they are distinct from one another. Research asks a specific question about an unknown aspect of medical science, designs an environment to answer that question, and tests the hypothesis within a specific environment that is meant to be applied generally. QI looks at what we know and applies it to the healthcare system already in existence. Because of this difference, research is designed to produce generalizable knowledge while QI is primarily interested in in the change of an individual system. Another key difference is the time frame for both of these activities. Research stops when it answers its specific question. QI is a continuous process that refines itself continuously with multiple cycles of Improvement.^41,42^

1. What responsibilities to patients do physicians have when practicing Improvement?

Both Improvement and HSR can pose a risk to patients by utilizing patient healthcare information.^41^ This could allow for unintentional disclosure. QI has the added risk that it can involuntarily constrain practice for the individual provider without informed consent which is typically not obtained during QI. The benefits of Improvement outweigh these potential risks and is generally accommodated by healthcare organizations such that institutional review is not required.^41^ However, it is critical that physicians realize that their improvement work could negatively impact patients. Physicians are obligated to monitor the changes to system for negative effects. Any systematic change in practice and evaluation should be considered by an institutional review board to determine if the goal of the activity is generalizable knowledge and if additional patient protections are warranted.^43,44^

You have created a team of stakeholders interested in decreasing the number of patients with prolonged PACU stays consisting of other anesthesia providers, nurses from the PACU, and a hospital level administrator. None of the members of the team have completed a QI project but they all assure you that they are eager to help in any way they can. You wonder, “Where do I even begin?”

1. What are the stages of a QI project?

There are numerous ways to approach QI. Less important than the choice to use a specific model for QI is moving forward with the project!^13,22-24,45^ Most approaches to QI incorporate cycles such as Plan-Do-Study-Act (PDSA) to frame each phase of the project.^12,46^ The repetitive nature of these steps stress that the initial plan or intervention will not necessarily be final improvement within the system. Improvement is continuous, and as we learn more and more about the system changes will be needed to refine any progress further.^22-24,45^ The phases of QI are innovation or coming up with new ideas for change, piloting those changes or testing on a small scale, implementing the change by making it standard in a certain setting, and spreading the changes to other settings. While these phases usually take place generally in order, it is common to return to a previous stage as you clarify the system and further refine an intervention. Depending on the expertise and model used by those performing QI, these phases can look very different. However, repetitive PDSA cycles within this general framework is usually a reasonable starting point for QI beginners.^23,47^

A key starting point for developing an intervention to improve your system is a clear understanding of the realities that impact the system as it currently exists. In particular the factors which cause variation in outcomes in the system are important. In healthcare the cause for variation can be very complex and difficult to explain in a concise manner. While it may not be critical to exhaustively understand all of these factors, it is important to gain an understanding of what you can control to make the system change. This understanding can only be achieved by observing the system and investigating the reasons it produces the outcomes it does. This often requires input from personnel with a firsthand understanding of the system.^6,22,23^

After examining the system, it is important to set goals for the project. What will improvement look like? This gives clear direction to the path we take to improvement and anchors the direction of our QI project to a specific outcome.^23,46^ Using frameworks such as the SMART goal criteria can be helpful in developing these outcome measurements. This suggests that goals for QI should be specific, measurable, attainable, relevant to patients, and time basaed.^48^ Not using these criteria can lead to confusion about the ultimate purpose for the QI project. Once we know what we want improvement to look like we must decide what intervention will lead to that measurable goal. In most cases interventions should start small. A test group that the intervention can be easily applied to and in whom the outcomes can be easily observed is often most appropriate. It is possible that the findings from this pilot intervention will lead to a greater understanding of the system and additional refinement of the goal or intervention will be necessary. Understanding the test sample and how it relates to the overall system being changed is key to appreciating if the results from this test group reflect the likely results when applying the intervention to the larger system being improved.^49^

Spread is critical to getting the improvement to the people that need it. But it is not always easy to spread the valuable intervention even after you have shown it works. The advantage of a change must be apparent to those implementing it. This is another reason why involving those who work within the system being improved will help reach meaningful improvements. Changes that are simple and compatible with work flow are the easiest to adopt. This often requires partnerships with numerous stakeholders who have an interest in seeing the improvement take hold.^23,50^

The department chief wants an update on your progress. Given the complexity of your system you are struggling with how to present you progress in a concise manner.

1. How can an A3 template help to communicate your project’s progress?

Many tools and systems for QI exist. One tool used to communicate QI projects in a concise manner that is very intuitive for those familiar with the scientific method is the A3.^51^ It allows for tracking of changes from the current to the desired state of Improvement. The name is derived from the size of the paper the chart is typically created on.^52^ The A3 template consists of 10 sections that can be adapted to the system that is being addressed. These sections allow to track the overall progress as the projects multiple PDSA cycles progress. A key benefit of this template is that it allows an understanding of the Improvement on a single piece of paper.^51,53^

Now that we have outlined the purpose and process of QI for anesthesiologists, let’s apply some of these concepts and tools. We will work through our PACU problem.

1. What do you want to know about the system you are improving?

Learners should ask if a pattern exists in terms of certain operating rooms or personnel that are responsible for a large proportion of patients with PACU stays greater than 3 hours. It is also important to understand the characteristics of long PACU stays. What is the average amount of time the patients spend in the PACU? Are patients that have particular types of surgery at a higher risk for prolonged PACU stays? These types of questions are critical for the first three steps of the A3 template. In these steps the issue to be improved is identified and the background information that is relevant to understand the system as it currently exists is gathered and organized.

Investigation of long PACU stays providers the following information over the course of the month:

| Number of PACU recoveries | Average PACU Stay (hrs) | Number of stays over 3 hours | Proportion of PACU Stays over 3 hours | Proportion of prolonged stay for pain | Proportion of prolonged stay for nausea | Proportion of prolonged stay for sedation |
| --- | --- | --- | --- | --- | --- | --- |
| 1156 | 2.1 | 347 | 0.3 | 0.05 | 0.69 | 0.26 |

There does not appear to be a pattern to the prolonged stays in terms of the type of surgical procedure or the involved personnel. Based on this info you feel that a large proportion of patients who have a prolonged PACU stay are due to postoperative nausea and vomiting (PONV).

1. Develop a concise the goal for improvement, describe the root cause of the current state, and define the target condition for improvement.

The learners here should resist the urge to define Improvement for this project as a decrease in PONV. The original goal of this project was to decrease the number of patients that require a PACU stay greater than 3 hours. Learners should keep in mind the SMART goal criteria.^48^ While patients certainly care about PONV, individual patient experience can be difficult to measure and compare. A goal of less than 20% of patients requiring a PACU stay of more than 3 hours within 2 weeks of the intervention is appropriate. It may seem obvious that 20% of patients remaining in the PACU for more than 3 hours is undesirable as a final state. However, it is important for the learners to realize that initial goals should be attainable. This will demonstrate improvement which can help with buy in as QI efforts are expanded in subsequent cycles of the project.

Further analysis into PONV in this group yielded the following information:

| Number of PACU recoveries > 3 hrs | Proportion of patients receiving inhalational anesthesia | Proportion of patients receiving one antiemetic in OR | Proportion of patients receiving two antiemetics in OR | Cost in antiemetic(s) per patient |
| --- | --- | --- | --- | --- |
| 347 | 0.98 | 0.72 | 0.23 | $8.96 |

Based on this analysis your team decides to add pre and postoperative PONV order sets with selectable medications for prophylaxis and treatment to the electronic health record.

1. Develop an intervention. Outline the implementation and follow up for this intervention.

Learners should define a plan for the implementation of this countermeasure. It is important to define who will be included in this pilot group, who will be ordering any premedication (anesthesiologist or surgeon), when will the patient receive premedication, who will track the proportion of these patients that have PACU stays more than 3 hours, and how long the pilot will occur before reanalysis. The specifics of this discussion are not as important as the application of the concepts of QI.

Your team gathered the following results for the 2-week time period for the pilot group:

| Proportion of PACU stays > 3 hours | Proportion of patients receiving one antiemetic | Proportion of patients receiving two antiemetics | Cost in antiemetic(s) per patient |
| --- | --- | --- | --- |
| 0.19 | 1.0 | 1.0 | $111.29 |

Just then you get a call from the department chief for what you assume will be a thank you for decreasing the proportion of PACU stays longer than 3 hours. Instead, she is very irate. Pharmacy is breathing down her neck because the cost of antiemetics used by the department in the last 2 weeks has spiked. Anesthesia providers seem to be premedicating more patients with aprepitant (which was included in your order sets) lately.

1. Is a QI project that does not achieve the desired end state a failure? Once a pilot group has encountered an unexpected problem, what is the next step?

Cost is an important factor in QI. It is possible that cost will make an intervention untenable within a particular system. However, just because the first solution proved to be too expensive for the system to tolerate does not mean that the project is a failure. Valuable information was learned from this work. Namely that it is possible to decrease the proportion of PACU stays longer than three hours by addressing PONV. This reinforces the initial root cause determination. Once an unexpected problem has occurred with a chosen solution it is time to return to the PDSA cycle. In these successive PDSA iterations it is important to consider what was learned in previous cycles. In this case the overall cost of the system may be highlighted. The decreased cost of late running ORs may offset the increased cost of antiemetic medications. Taking only the pharmacy or PACU view of the system does not allow for overall system improvement. It is important to analyze the changes seen and use them to plan future interventions that further improve the system. In this way the scientific method is integral to the process of QI.

Learners should develop new questions that will increase with their understanding of the system. They also should create changes to their countermeasure which may allow for improvement with acceptable cost. These could include risk stratification of patients for PONV to use aprepitant in only the highest risk, maximizing antiemetics other than aprepitant, and limiting the use of inhaled anesthetics and opioids.

The views expressed in this problem based learning discussion are those of the authors and do not reflect the official policy of the Department of Defense or the U.S. Government.

References

1. Zeidel ML. Systematic quality improvement in medicine: everyone can do it. *Rambam Maimonides Med J*. 2011;2(3): e0055. https://doi.org/10.5041/rmmj.10055.
2. Kohn LT, Corrigan JM, Donaldson MS. Eds. To Err Is Human: Building a Safer Healthcare System. Washington, D.C: Institute of Medicine, National Academy Press; 2000. https://doi.org/10.17226/9728.
3. Swensen SJ, Meyer GS, Nelson EC, et al. Cottage industry to postindustrial care – the revolution in healthcare delivery. *N Engl J Med*. 2010;362: e12. https://doi.org/10.1056/nejmp0911199.
4. Zeidel ML, James BC. Improving the quality of healthcare in America: what medical schools, leading medical journals, and federal funding agencies can do. *Am J Med.* 2002;112:165–7. https://doi.org/10.1016/s0002-9343(01)01099-3.
5. Deming WE. 2nd ed. Cambridge, MA: IT Press; 2000. The New Economics for Industry, Government, Education.
6. Womack JP, Jones DT, Roos D. New York, NY: Rawson Associates, McMillan Publishing; 1990. The Machine That Changed the World.
7. Fleisher LA. Quality Anesthesia: Medicine Measures, Patients Decide*.* *Anesthesiology*. 2018; 129:1063-9. https://doi.org/10.1097/ALN.0000000000002455.
8. Khuri SF, Daley J, Henderson W, et al. The Department of Veterans Affairs’ NSQIP: The first national, validated, outcome-based, risk adjusted, and peer-controlled program for the measurement and enhancement of the quality of surgical care. Ann Surg. 1998; 228: 491–507. https://doi.org/10.1097/00000658-199810000-00006.
9. Finks JF, Osborne NH, Birkmeyer JD. Trends in hospital volume and operative mortality for high-risk surgery. N Engl J Med. 2011; 364: 2128–37. https://www.nejm.org/doi/full/10.1056/NEJMsa1010705.
10. Dutton RP. Ethical questions in anesthesiology. AMA J Ethics. 2015; 17(3): 248-52. https://doi.org/10.1001/journalofethics.2015.17.3.pfor1-1503.
11. Wagstaff DT, Bedford J, Moonesinghe SR. Improvement Science in Anaesthesia. *Curr Anesthesiol Rep*. 2017; 7(4): 432‐9. https://doi.org/10.1007/s40140-017-0234-5
12. Silver SA, Harel Z, McQuillan R, et al. How to Begin a Quality Improvement Project. *Clin J Am Soc Nephrol*. 2016; 11(5): 893-900. https://doi.org/10.2215/CJN.11491015
13. Taitz JM, Lee TH, Sequist TD. A framework for engaging physicians in quality and safety. *BMJ Qual Saf*. 2012; 21(9): 722–8. https://doi.org/10.1136/bmjqs-2011-000167
14. Audet AM, Doty MM, Shamasdin J, et al. Measure, learn, and improve: Physicians’ involvement in quality improvement. *Health Aff (Millwood)*. 2005; 24(3): 843–53. https://doi.org/10.1377/hlthaff.24.3.843
15. Pronovost PJ, Miller MR, Wachter RM, et al. Perspective: Physician leadership in quality. *Acad Med*. 2009; 84(12): 1651–6. https://doi.org/10.1097/acm.0b013e3181bce0ee.
16. Lindenfeld S, Vlchek D: Engaging physicians in continuous quality improvement. *Adv Ren Replace Ther*. 2001; 8(2): 120–4. https://doi.org/10.1053/jarr.2001.23985.
17. Berwick DM. The science of improvement. *JAMA.* 2008; 299(10): 1182–4. https://doi.org/10.1001/jama.299.10.1182
18. Committee on Quality Health Care in America IoM: Crossing the Quality Chasm: A New Health System for the 21^st^ Century. Washington, D.C., National Academies Press, 2001. https://doi.org/10.17226/10027
19. Miller TE, Mythen M. Successful recovery after major surgery: moving beyond length of stay. *Perioper Med (Lond).* 2014; 3: 4. Published 2014 Jul 8. https://doi.org/10.1186/2047-0525-3-4
20. Peden CJ, Campbell M, Aggarwal G. Quality, safety, and outcomes in anaesthesia: what's to be done? An international perspective. *Br J Anaesth.* 2017; 119(suppl_1): i5‐i14. https://doi.org/10.1093/bja/aex346
21. Institute for healthcare improvement Open school. QI 101: Introduction to healthcare improvement. http://app.ihi.org/lmsspa/#/6cb1c614-884b-43ef-9abd-d90849f183d4/60967fa6-4642-4f33-9ec2-60083d52d0fe. Accessed 12 June 2020.
22. Langley GL, Moen R, Nolan KM, Nolan TW, Norman CL, Provost LP. The Improvement Guide: A Practical Approach to Enhancing Organizational Performance. 2nd ed. San Francisco, California: Jossey-Bass Publishers; 2009.
23. Ogrinc GS, Headrick LA, Moore SM, et al. Fundamentals of Health Care Improvement: A Guide to Improving Your Patient’s Care, Cambridge, MA, Institute for Healthcare Improvement, 2012.
24. Pukenas EW, Patel K. Performance improvement to address anesthesia hazards. *Int Anesthesiol Clin*. 2020; 58(1): 38‐44. https://doi.org/10.1097/AIA.0000000000000263
25. Stoelting RS. Patient safety: a brief history. In: Ruskin K, Stiegler M, Rosenbaum S, eds. Quality and Safety in Anesthesia and Perioperative Care. New York, NY: Oxford University Press; 2016:3–15
26. Dutton RP, Dukatz A. Quality improvement using automated data sources: the anesthesia quality institute. *Anesthesiol Clin.* 2011; 29(3): 439–54. https://doi.org/10.1016/j.anclin.2011.05.002
27. Registries, NACOR, Intro to NACOR https://www.aqihq.org/introduction-to-nacor.aspx. Accessed 12 June 2020.
28. Steadman RH, Berry AJ, Coursin DB, et al. Simulation and MOCA^®^: ASA and ABA perspective, after the first three years. ASA Monitor. 2013; 77(8): 30-2. https://monitor.pubs.asahq.org/article.aspx?articleid=2431638. Accessed 12 June 2020.
29. McIvor W, Burden A, Weinger MB, et al. Simulation for maintenance of certification in anesthesiology: the first two years. *J Contin Educ Health Prof*. 2012; 32(4): 236‐42. https://doi.org/10.1002/chp.21151.
30. Specialties, Anesthesiology, Program Requirements and FAQs. https://www.acgme.org/Specialties/Program-Requirements-and-FAQs-and-Applications/pfcatid/6/Anesthesiology. Accessed 12 June 2020.
31. Institute for healthcare improvement Open school. QI 201 Improvement takes Practice 201: Why engage trainees in quality and safety? http://app.ihi.org/lmsspa/#/6cb1c614-884b-43ef-9abd-d90849f183d4/960820a0-d9ed-4bce-9e95-bddbd30142d0. Accessed 1 June 2020.
32. Kalanithi L, Coffey CE, Mourad M, et al. The effect of a resident-led quality improvement project on improving communication between hospital-based and outpatient physicians. American Journal of Medical Quality. 2013; 28: 472-9. https://doi.org/10.1177/1062860613478976.
33. Kurth CD. Introducing quality improvement. *Paediatr Anaesth.* 2013; 23(7) :569–70. https://doi.org/10.1111/pan.12167.
34. Kurth CD, Tyler D, Heitmiller E, et al. National pediatric anesthesia safety quality improvement program in the United States. *Anesth Analg*. 2014; 119(1): 112‐121. https://doi.org/10.1213/ANE.0000000000000040
35. Haller G, Stoelwinder J, Myles PS, et al. Quality and safety Indicators in anesthesia: A systematic review. *Anesthesiology*. 2009; 110: 1158–75. https://doi.org/10.1097/ALN.0b013e3181a1093b.
36. Hyder JA, Hebl JR. Performance measurement to demonstrate value. *Anesthesiol Clin*. 2015; 33: 679–96. https://doi.org/10.1016/j.anclin.2015.07.007.
37. Jankowski CJ, Walsh MT. Quality Improvement in Ambulatory Anesthesia: Making Changes that Work for You. *Anesthesiology Clin*. 2019; 37: 349-60. https://doi.org/10.1016/j.anclin.2019.01.013.
38. Patient-Centered, Value based Health Care is Incompatible With the Current Climate of Excessive Regulation, “Health Affairs Blog, October 3, 2019. https://www.healthaffairs.org/do/10.1377/hblog20180927. Accessed 3 May 2020.
39. Caplan RA, Posner K, Ward RJ, et al. Peer Review agreement for major anesthetic mishaps. *Quality Review Bulletin.* 1988; 14: 363-8. https://doi.org/10.1016/s0097-5990(16)30248-2
40. Goldman R. The reliability of peer assessments of quality of care. *JAMA.* 1992; 267: 958-60. https://doi.org/10.1001/jama.1992.03480070074034
41. Stiegler MP, Tung A. Is It Quality Improvement or Is It Research?: Ethical and Regulatory Considerations. *Anesth Analg.* 2017; 1 25(1): 342‐4. https://doi.org/10.1213/ANE.0000000000001815.
42. O'Neill SM, Hempel S, Lim YW, et al. Identifying continuous quality improvement publications: what makes an improvement intervention 'CQI'?. *BMJ Qual Saf*. 2011; 20(12): 1011‐9. https://doi.org/10.1136/bmjqs.2010.050880
43. Bellin E, Dubler NN. The quality improvement-research divide and the need for external oversight. *Am J Public Health*. 2001; 91(9): 1512–7. https://doi.org/10.2105/ajph.91.9.1512
44. Nerenz DR, Stoltz PK, Jordan J. Quality improvement and the need for IRB review. *Qual Manag Health Care*. 2003; 12: 159–70. https://doi.org/10.1097/00019514-200307000-00006
45. Institute for healthcare improvement open school. QI: 102: How to improve with the model for improvement. http://app.ihi.org/lmsspa/#/6cb1c614-884b-43ef-9abd-d90849f183d4/41b3d74d-f418-4193-86a4-ac29c9565ff1/lessonDetail/788b1b5e-de97-4d8a-ac8c-fe4bd61f397a/page/2. Accessed 22 June 2020
46. Moonesinghe SR, Peden CJ. Theory and context: putting the science into improvement. *Br. J. Anaesth*. 2017; 118(4) :482–4. https://doi.org/10.1093/bja/aew469.
47. Institute for healthcare improvement open school. QI: 105: Leading Quality Improvement. http://app.ihi.org/lmsspa/#/6cb1c614-884b-43ef-9abd-d90849f183d4/6d2b36c7-eee1-41b4-a852-67d1c4837404/lessonDetail/176bda31-d020-4189-b4eb-05033d6183bd/page/0. Accessed 22 June 2020
48. Doran GT. There's a S.M.A.R.T. way to write management's goals and objectives. *Manage Rev*. 1981; 70: 35–6.
49. Institute for healthcare improvement open school. QI: 103: Testing and Measuring Changes with PDSA cycles. http://app.ihi.org/lmsspa/#/6cb1c614-884b-43ef-9abd-d90849f183d4/7ab177dc-a9cf-4d1d-b870-f4be6e8d8f67/lessonDetail/7ea95efc-454f-44a9-a0d0-b70a0152e1e8/page/2. Accessed 22 June 2020.
50. Institute for healthcare improvement open school. QI: 201: Planning for Spread: From Local Improvements to system wide change. http://app.ihi.org/lmsspa/#/6cb1c614-884b-43ef-9abd-d90849f183d4/ea07c796-a771-4713-8bd8-520188b6c793/lessonDetail/26bb0d17-0992-4a05-afd9-e77f842bcc5a/page/6. Accessed 22 June 2020
51. Bassuk JA, Washington IM. The A3 problem solving report: a 10-step scientific method to execute performance improvements in an academic research vivarium. *PLoS One*. 2013; 8(10) :e76833. Published 2013 Oct 29. https://doi.org/10.1371/journal.pone.0076833
52. Anderson JB, Marstiller H, Shah K. Lean Thinking for Primary Care. *Prim Care*. 2019; 46(4): 515-27. https://doi.org/10.1016/j.pop.2019.07.009
53. Sobeck DK. Steps of the A3 process. Montana State University. Mechanical and Industrial Engineering. http://www.montana.edu/dsobek/a3/steps.html. Accessed 24 June 24, 2020.
