# Supplementary material for: Problem-Based Learning Discussion to Introduce Quality Improvement to Residents in the Perioperative Setting
Source: MedEdPORTAL. 2021 Nov 29;17:11198. doi: 10.15766/mep_2374-8265.11198 (PMC8627916; doi:10.15766/mep_2374-8265.11198)
Supplement: Supplementary file 1 — Staff Feedback Questionnaire.docxPre-PBLD Learner Survey.docxCase Stem and Required Reading.docxPost-PBLD Learner Survey.docxModel Learning Discussion.docx [file mep_2374-8265.11198-s001.zip › A. Staff Feedback Questionnaire.docx]

**Staff Anesthesiologist Feedback questions**:

1. What was your overall impression of the session? Positive/negative?
2. What parts of the session will help you improve your ability to complete a quality improvement project? What parts were not helpful? Why?
3. Is this a good way to teach residents about Improvement?
4. What is the primary thing you would change about the PBLD to make it better?
