# Supplementary material for: Problem-Based Learning Discussion to Introduce Quality Improvement to Residents in the Perioperative Setting
Source: MedEdPORTAL. 2021 Nov 29;17:11198. doi: 10.15766/mep_2374-8265.11198 (PMC8627916; doi:10.15766/mep_2374-8265.11198)
Supplement: Supplementary file 1 — Staff Feedback Questionnaire.docxPre-PBLD Learner Survey.docxCase Stem and Required Reading.docxPost-PBLD Learner Survey.docxModel Learning Discussion.docx [file mep_2374-8265.11198-s001.zip › Educational_Summary_Report_11198.pdf]

MedEdPORTAL® The AAMC Journal of Teaching and Learning Resources

Original Publication

Open Access

Problem-Based Learning Discussion to Introduce Quality Improvement to Residents in the Perioperative Setting

Benjamin M. Kristobak, MD*, Jesse A. Snider, MS, DO

*Corresponding author: kristobak.ben@gmail.com

Abstract

Introduction: Quality improvement (QI) is a growing and critical part of perioperative medical practice. However, there are few examples of educational tools to introduce new learners from anesthesiology to QI. This may contribute to a lack of enthusiasm to learn about and apply these concepts. **Methods:** This problem-based learning discussion (PBLD) was designed to teach anesthesiology residents about QI in a way allowing for the application of core concepts in a group setting. We created this PBLD using available literature on QI in the perioperative setting. Basic concepts and terminology necessary for new learners to communicate about QI were specifically addressed. Feedback from staff anesthesiologists and resident participants in the PBLD was used to tailor it to the needs of the target learners and to reach the educational objectives. **Results:** We delivered this PBLD in two separate learning sessions both to board-certified anesthesiologists ($N = 10$) and to resident anesthesiologists ($N = 19$) at our institution. The exercise was reviewed anonymously, and qualitative feedback was used to improve updated versions. Respondents felt that the PBLD would be improved by avoiding jargon-based humor, considering the systemic implications of QI, and limiting the overall length of the learning tool. The PBLD has been adopted as a starting point for discussions about QI in our training program. **Discussion:** We feel this PBLD can introduce new learners to the learning objectives. This tool has provided an alternative to lectures or computer-based modules for teaching QI.

Keywords
Anesthesiology, Curriculum Development, Quality Improvement/Patient Safety, Problem-Based Learning, Qualitative Research, Editor's Choice

Educational Objectives

By the end of this activity, learners will be able to:

1. State the purpose and value of improvement in the health care setting.
2. Describe the differences between patient safety, quality improvement, and quality assurance.
3. Describe the risks and responsibilities common in health care improvement.
4. List the phases of a quality improvement project.
5. Apply quality improvement concepts to a perioperative scenario.

Citation:
Kristobak BM, Snider JA. Problem-based learning discussion to introduce quality improvement to residents in the perioperative setting. *MedEdPORTAL*. 2021;17:11198.
https://doi.org/10.15766/mep_2374-8265.11198

Introduction

It is critical for physicians to understand and participate in quality improvement (QI). Doing so allows them to address health care disparities and can improve the quality of care provided throughout an individual's career.[1,2] The Accreditation Council for Graduate Medical Education (ACGME) has recognized this and made QI a mandatory component of graduate medical education (GME) curricula across specialties.[3] As a specialty, anesthesiologists have recognized the importance of QI in generating better patient outcomes in the perioperative setting.[4,5] The American Board of Anesthesiology has made participation in QI mandatory for maintenance of certification,[6] and the American Society of Anesthesiologists (ASA) created the Anesthesia Quality Institute to facilitate the tracking and use of quality measures among anesthesiologists.[7,8]

The majority of resources to teach resident physicians about QI concepts were developed for training programs in internal medicine and pediatrics[9-16]; however, *MedEdPORTAL* also contains examples for trainees in obstetrics and gynecology[17]

Copyright © 2021 Kristobak and Snider. This is an open-access publication distributed under the terms of the Creative Commons Attribution license.

and psychiatry.[18] Simulation may be emerging as a leading teaching modality for QI, but so far, published examples are limited to scenarios created for psychiatry[19] and pediatrics.[20] These simulations require expertise, time, and resources to implement. There are limited examples of QI curricula designed for trainees in surgical specialties,[17,21,22] but we are not aware of any open-access published tools designed specifically for anesthesiology residents. Some GME program faculty may feel unprepared to teach QI due to lack of expertise in teaching or applying QI concepts, especially if curricula have not previously been developed for their medical specialty.

Problem-based learning (PBL) methodologies have been well integrated into the continuing medical education of anesthesiology. This is perhaps because the practice of anesthesiology requires real-time, time-limited data interpretation along with creative reasoning and critical thinking. The group format of PBL allows learners to develop these skills by generating discussion.[23] While there are several types of PBL learning, case-based discussions are probably the most recognizable among anesthesiologists.[24,25] These PBL discussions (PBLDs) have been a prominent part of every ASA annual meeting for decades.[24] PBL allows group learning between peers[26] without sacrificing the quality of learning if a subject matter expert is not available.[27] For these reasons and because of work demonstrating the benefits of a flipped classroom in teaching residents about QI,[28] we feel that a PBLD is an optimal means to teach anesthesiology residents about QI.

We developed a PBLD activity applying terminology and methods recognizable in the daily workflow of anesthesiologists to address the needs of anesthesiology GME training programs. We feel this application assists in grasping QI concepts. Training applicable to anesthesiologists that can be taught by group discussion facilitated by nonexpert instructors is likely of value to many anesthesiology training programs.

Methods

Prior to this PBLD, a lack of resources and education in QI was cited as an area for improvement on our annual program ACGME survey for 2 consecutive years. In response, we created our PBLD to allow novices unfamiliar with the purpose, benefits, and basic differences between improvement activities to apply the basic concepts of QI. We designed our PBLD by literature review of QI in the perioperative setting and our own personal experience.

We first presented an initial version of the PBLD to anesthesiologists in our department with varying QI experience. After this session, we provided an early version of the learning discussion to these anesthesiologists for additional review. We asked four follow-up questions to elicit narrative feedback (Appendix A) and then incorporated these recommendations for improvement into revisions of the PBLD. Furthermore, we sought additional resources to address the anesthesiologists' concerns and to focus the applicability of the PBLD to perioperative experiences.

We asked anesthesiology residents in our program to complete a survey (Appendix B) to determine their enthusiasm for learning about QI and their confidence in being able to complete a project. After this, we provided the PBLD case stems and required reading (Appendix C) consisting of open-access journal articles[4,29,30] to be read by learners before taking part in the PBLD. (In order to ensure that open-access and enduring materials can be supplied for future iterations of this PBLD, we have here updated these required reading materials from the original ones selected.) We also provided an exhaustive list of resources that informed the learning discussion to the resident learners at this time, but these were not required reading.

We conducted the resident PBLD at an afternoon academic session with learners both in person and using tele- and videoconferencing. This was necessary because of restrictions in response to the COVID-19 pandemic. The facilitator explained the framework of the PBLD session and reminded the learners that they would be leading the discussion. The facilitator would only speak if needed to help the learners verbalize the answers to questions and case scenarios. The learners were then asked to read the learning objectives and encouraged to ask any clarifying questions about the framework of the PBLD. Next, the facilitator read the case stem and the first question for discussion aloud. The learners began to discuss the answer to the question. The facilitator listened to the discussion and, if necessary, allowed periods of silence to encourage the learners to continue their discussion. If the learners did not verbalize a key point, the facilitator would ask a question to the group to generate discussion along those lines. The facilitator also directed questions to encourage comment from learners who made fewer contributions to the group discussion. This process continued for each question. Throughout, the facilitator served as a resource to help the learners verbalize the key concepts in the learning discussion and the learning objectives.

After the learning session, we provided residents with another survey based on a learner feedback survey used by Greenlaw, Jacob, and Cheston[20] (Appendix D). We recorded mean Likert scores and standard deviations of the answers on this survey as a surrogate for residents' interest in QI and their opinion

Copyright © 2021 Kristobak and Snider. This is an open-access publication distributed under the terms of the Creative Commons Attribution license.

on the effectiveness of the tool. Learners were also asked to provided narrative comments about the PBLD. We shared the model learning discussion (Appendix E) with learners at this time because it was convenient to do so and we felt that learners might want to review selected areas of the model learning discussion on which they wished to give feedback. Based on survey results and narrative feedback provided by learners, we made additional improvements to the PBLD stem, questions, and model learning discussion.

We designed the evaluation of the PBLD to obtain qualitative, narrative feedback from both sessions. The goal of obtaining this feedback was to further improve the resource for future use. We included a resident survey simply to ascertain the overall feelings about this resource. During the resident PBLD session, we gauged and made notes about resident participation.

Results

The initial PBLD session presented to 10 board-certified anesthesiologists in our department took 90 minutes to complete. The first portion of the PBLD clarifying the types of improvement and the steps of QI took 60 minutes to complete. The second part utilizing the case example took 30 minutes. Eight of the 10 participants had reviewed the suggested resources prior to the PBLD.

Narrative feedback was supportive of the learning tool and indicated that it was an effective way to introduce new learners to QI. Examples of positive feedback included the following:

- “[Overall] my impression is very positive. This is a comprehensive introduction.”
- “This is a critically important topic. Creating a forum to discuss the basics and to establish a common language [for that dialogue to occur] is extremely valuable.”
- “Good discussion, [which leads to] a good debate.”
- “Great intellectual discussion that is also applicable to the practice environment.”

However, other comments highlighted shortcomings with the original narrative and made suggestions for improvement. Several staff did not like the way that the original version of the PBLD used cynical, jargon-based humor to seemingly disparage the efforts of both physicians and nonphysicians to improve patient care. Comments included the following:

- “The case has some negative images of physicians. Consider a more positive approach to keep learners feeling positive about QI.”
- “Anesthesiologists don’t ‘Just want to do their job and go home,’ and I don’t think ‘No good deed goes unpunished.’ How about something in a more positive mode?”

Other staff highlighted the importance of systemic factors in addressing QI. In particular, the importance of defining appropriate outcomes and of observing those changes was stressed. These respondents felt learners ought to grasp that system change should matter to patients and be supported by scientific evidence and that the system should be monitored for unexpected changes that could negatively impact patient care.

- “[With regard to the scenario,] I would expand it to include additional elements that will help in defining the stakeholder, metrics, and outcomes that will be appreciated. The backup of ORs has costs to the system. Are we defining success for the system overall or just postoperatively?”
- “How can we get help to better address our patient’s needs? Building an effective team is important to solving any problem and understanding any system.”
- “This is a critically important topic. It is difficult to learn without a forum to discuss and a common language to discuss it in. I would consider linking this case to evidence-based practice guidelines and how they can help you understand [a] system.”

There were also concerns about the length of the original presentation. Developing a concise and effective approach to learning was critical for these anesthesiologists, who felt themselves getting lost or confused with the original version.

- “I would hammer home the message of patient centered care. I feel like that got a little lost in the push to show why quality is beneficial to organizations.”
- “This is probably a little too long to hold residents’ attention. I think we lost the ‘problem’ through the presentation.”

Thirty-one of our residency’s 51 residents (PGY 1-PGY 4) completed the pre-PBLD learner survey (Table 1). Results of this survey showed that residents had interest in learning about and utilizing QI. Also, these residents were more likely to say that they were confident in their skills and understanding of QI than may have been expected from the results of the program’s annual ACGME survey. Not all of these residents went on to complete the PBLD because many were at remote training sites on the day the PBLD learning session was held.

Copyright © 2021 Kristobak and Snider. This is an open-access publication distributed under the terms of the Creative Commons Attribution license.

Table 1. Pre-PBLD QI Survey (N = 31)

Question[a]	*M*	*SD*
I think learning about QI is an important part of learning to be an anesthesiologist.	3.7	1.1
Learning about QI will prepare me for success on in-training and board examinations.	3.1	1.1
I will use QI in my day to day practice as an anesthesiologist.	3.6	0.9
The only reason I will complete a QI project is because the Accreditation Council for Graduate Medical Education has made it a requirement.	3.4	1.4
I am confident that I can complete a QI project that will positively impact others.	3.7	1.0

Abbreviations: PBLD, problem-based learning discussion; QI, quality improvement.
[a]Rated on a 5-point Likert scale (1 = *strongly disagree,* 2 = *disagree,* 3 = *neutral,* 4 = *agree,* 5 = *strongly agree*).

Nineteen anesthesiology residents completed the PBLD. Nine residents were in person for the discussion; all others were connected using tele- and videoconferencing. Thirteen of these 19 residents reported having reviewed the suggested material to prepare for the PBLD. The PBLD took roughly 120 minutes to complete, including a 15-minute break at the midpoint.

After completing the PBLD, 14 residents filled out the post-PBLD survey (Table 2). As in the pre-PBLD learner survey, learners had a more confident view of their own understanding of QI prior to the learning session than we suspected before conducting these surveys. Still, learners responded that the activity provided a realistic scenario and a valuable experience. On average, they agreed that they were more confident in their skills in leading a QI initiative after completing the PBLD. The responses to understanding the subject matter for participation and the likelihood of starting a QI initiative were neutral on average.

Examples of positive qualitative feedback included the following:

- “I thought that the example simulation was a fantastic way to apply the principles [of QI].”

Table 2. Post-PBLD QI Survey (N = 14)

Question[a]	*M*	*SD*
Before the PBLD, I felt completely confident in my abilities to lead a QI initiative.	2.4	0.7
I understood the subject matter of the scenario well enough to actively participate.	3.0	0.4
The PBLD provided me with a realistic scenario with a reasonable progression.	4.2	0.6
I gained valuable experience in completing this PBLD.	3.9	0.6
After the PBLD, I feel more confident in my abilities to lead a QI initiative.	3.7	0.9
After the PBLD, I am more likely to start my own QI initiative.	3.1	0.9

Abbreviations: PBLD, problem-based learning discussion; QI, quality improvement.
[a]Rated on a 5-point Likert scale (1 = *strongly disagree,* 2 = *disagree,* 3 = *neutral,* 4 = *agree,* 5 = *strongly agree*).

- “I think this is a great way to teach and discuss QI, and most things in general.”
- “The discussion and debate were great.”

Several residents thought that this learning session could be expanded with more examples.

- “I almost wish we had done two scenarios instead of one.”
- “I think we could read about these concepts [on our own]. I wish we had more ways to practice this.”

As was the case with the staff anesthesiologists, efficiency was important to these learners. Several comments indicated that the learning discussion could be shortened and still reach the same learning objectives.

- “Content was great, perhaps too many different resources to parse through before PBLD.”
- “This was a lot to get through in one sitting. I am not sure the reading helped me prepare as much as talking with other residents.”

Residents were frustrated by the need to use video- and teleconferencing to perform a PBLD.

- “Somewhat hampered by virtual participation but overall a good intro to QI, [quality assurance], and [patient safety].”
- “In person would have been better.”

Discussion

To teach QI to anesthesiology residents, we developed a PBLD with a focus on topics specific to the perioperative setting. This methodology appears to be feasible, and our example speaks to its acceptability, implementation, and practicality, as well as its adaptability to the irregular circumstances of the COVID-19 pandemic.[31] This tool and ones like it could be an alternative QI curriculum to lectures or online modules by using scenario discussions that allow learners to teach one another. The learner who commented, “This was a lot to get through in one sitting. I am not sure the reading helped me prepare as much as talking with other residents,” highlighted the value of peer teaching and communication in acquiring the content knowledge of the learning objectives. Their comment supports the idea that collaboration and problem-solving skills are better addressed by group learning such as PBLDs in comparison to individual learning such as online modules.[23,26,28] It is also likely that our PBLD takes fewer resources and less time to teach these skills compared with QI simulations.[19,20]

The inclusion of staff anesthesiologists involved in the implementation and administration of patient care in the

Copyright © 2021 Kristobak and Snider. This is an open-access publication distributed under the terms of the Creative Commons Attribution license.

revisions of the case stem and learning discussion was critical in developing this PBLD. These experts highlighted the systemic nature of improvement and patient systems that may have been difficult for trainees to otherwise appreciate. They also emphasized the importance of modeling resident perceptions and attitudes about learning. While humor can help engage learners in an activity, overreliance on tropes could be difficult for trainees to interpret and be seen as promoting a lack of empathy for patients and colleagues.

Trainee survey responses were somewhat unexpected. Both pre- and post-PBLD learner survey responses suggested that residents had more baseline knowledge about QI than we expected based on our training program's annual ACGME survey. The inclusion of senior (PGY 3 and PGY 4) residents in our sample was likely responsible for some of this feedback. Residents in our program are required to complete an improvement or patient safety project during their training. Senior residents are more likely to have completed this project and thus have more expertise with these topics than junior (PGY 1 and PGY 2) residents. The narrative feedback from senior residents is likely still of value because they were able to evaluate whether this PBLD would have helped them learn about QI to complete their improvement/patient safety project. This supports our goal of producing a PBLD QI resource responsive to the needs of anesthesiology resident learners.

The need for off-site rotations because of the nature our institution limited the number of PBLD sessions possible. Some of these residents were able to participate via teleconference, but it was not feasible for all depending on a resident's current assignment. Therefore, while many residents were able to complete the internet-based prelearning survey, fewer were able to participate in the learning session. Also, because of the structure of our training program's curriculum, more senior residents were on outside rotations and therefore less able to participate in the learning session compared to junior residents (PGY 2s). This did not seem to have an effect on the likelihood of which residents would actively participate in the PBLD as both junior residents and the senior residents who were present commented on the learning objectives and answered questions. Facilitator encouragement of less vocal learners was effective for both in-person and remote learners. Some of these less vocal learners made thoughtful observations that helped to steer the discussion toward the learning objectives when encouraged to comment.

Our single learning session limits the ability to evaluate the effectiveness of our intervention to teach the concepts of QI with the same external validity as other published resources. The ability of our surveys to elicit accurate feedback has not been validated. However, given that the concerns that led to the development of this tool were derived from the ACGME survey, it may be reasonable to assume that anonymous survey responses would identify similar concerns about the ability of this PBLD to address its learning objectives.

In the future, the learning by residents from a PBLD QI curriculum should be evaluated with a validated tool such as the Assessment of Quality Improvement Knowledge and Skills[21] or the Quality Improvement Knowledge Assessment Tool.[10,22,23] Comparing residents who did and did not participate in a PBLD QI curriculum using these validated measures would likely determine the curriculum's effectiveness in relation to other educational methodologies. Additional PBLDs in a QI curriculum are probably needed to provide comprehensive learning as measured by these validated tools. Once developed, synchronous learning from nonexpert facilitators would be a valuable resource for many anesthesiology training programs. We look forward to collaboration with other educators to develop and implement more QI PBLD learning scenarios that will allow for more comprehensive evaluation of the effectiveness of this methodology in a broader range of anesthesiology residents.

Appendices

A. Staff Feedback Questionnaire.docx

B. Pre-PBLD Learner Survey.docx

C. Case Stem and Required Reading.docx

D. Post-PBLD Learner Survey.docx

E. Model Learning Discussion.docx

All appendices are peer reviewed as integral parts of the Original Publication.

Benjamin M. Kristobak, MD: Staff Anesthesiologist, Walter Reed National Military Medical Center; Assistant Professor, Department of Anesthesiology, Uniformed Services University of the Health Sciences F. Edward Hébert School of Medicine; Associate Program Director, National Capital Consortium Residency in Anesthesiology; ORCID: https://orcid.org/0000-0003-4517-447X

Jesse A. Snider, MS, DO: Resident, National Capital Consortium Residency in Anesthesiology

Copyright © 2021 Kristobak and Snider. This is an open-access publication distributed under the terms of the Creative Commons Attribution license.

Acknowledgments
We appreciate the expertise and guidance of the *MedEdPORTAL* reviewers and editors. Their comments were able to develop this Educational Summary Report into a more meaningful and accurate account of methods that we used to evaluate and improve upon our problem-based learning discussion. Their expertise highlighted the future directions that will further develop tools like this one for anesthesiology residents. We greatly appreciate the opportunity to learn from and collaborate with these experts to create learning tools for anesthesiology trainees.

Disclosures
None to report.

Funding/Support
None to report.

Ethical Approval
Reported as not applicable.

Disclaimer
The views expressed in this publication are those of the authors and do not reflect the official policy of the Department of Defense or the U.S. Government.

References

1. Zeidel ML. Systematic quality improvement in medicine: everyone can do it. *Rambam Maimonides Med J*. 2011;2(3):e0055. https://doi.org/10.5041/rmmj.10055
2. Wong BM, Etchells EE, Kuper A, Levinson W, Shojania KG. Teaching quality improvement and patient safety to trainees: a systematic review. *Acad Med*. 2010;85(9):1425-1439. https://doi.org/10.1097/ACM.0b013e3181e2d0c6
3. Accreditation Council for Graduate Medical Education. *ACGME Common Program Requirements (Residency)*. Accreditation Council for Graduate Medical Education; 2020. Accessed November 5, 2020. https://www.acgme.org/Portals/0/PFAssets/ProgramRequirements/CPRResidency2020.pdf
4. Fleisher LA. Quality anesthesia: medicine measures, patients decide. *Anesthesiology*. 2018;129(6):1063-1069. https://doi.org/10.1097/ALN.0000000000002455
5. Jankowski CJ, Walsh MT. Quality improvement in ambulatory anesthesia: making changes that work for you. *Anesthesiol Clin*. 2019;37(2):349-360. https://doi.org/10.1016/j.anclin.2019.01.013
6. Quality improvement. American Board of Anesthesiology. Accessed September 13, 2021. https://theaba.org/about%20moca%202.0.html#qi%20section
7. About us. Anesthesia Quality Institute. Accessed June 17, 2019. https://www.aqihq.org/about-us.aspx
8. *2019 NACOR User Guide*. Anesthesia Quality Institute; 2019. Accessed December 5, 2019. https://www.aqihq.org/files/MIPS/2019/2019_NACOR_User_Guide_FINAL_OCT_2019.pdf
9. Stewart D, Lye C, Lopez M, Mothner B, Camp E, Vachani J. Engaging learners through modules in quality improvement and patient safety. *MedEdPORTAL*. 2016;12:10482. https://doi.org/10.15766/mep_2374-8265.10482
10. Keefer P, Orringer K, Vredeveld J, Warrier K, Burrows H. Developing a quality improvement and patient safety toolbox: the curriculum. *MedEdPORTAL*. 2016;12:10385. https://doi.org/10.15766/mep_2374-8265.10385
11. Werner JA. An integrated, multimodal resident curriculum in patient safety and quality improvement. *MedEdPORTAL*. 2017;13:10641. https://doi.org/10.15766/mep_2374-8265.10641
12. Cioletti A, Sweidan S. A joint quality improvement and high-value care curriculum in a limited-resource setting. *MedEdPORTAL*. 2017;13:10545. https://doi.org/10.15766/mep_2374-8265.10545
13. Dulay M, Saxe JM, Odden K, et al. Promoting quality improvement in primary care through a longitudinal, project-based, interprofessional curriculum. *MedEdPORTAL*. 2020;16:10932. https://doi.org/10.15766/mep_2374-8265.10932
14. Moses J, Shore P, Mann KJ. Quality improvement curricula in pediatric residency education: obstacles and opportunities. *Acad Pediatr*. 2011;11(6):446-450. https://doi.org/10.1016/j.acap.2011.08.007
15. Yanamadala M, Hawley J, Sloane R, Bae J, Heflin MT, Buhr GT. Development and assessment of a web-based clinical quality improvement curriculum. *J Grad Med Educ*. 2014;6(1):147-150. https://doi.org/10.4300/JGME-D-13-00140.1
16. Massagli TL, Zumsteg JM, Osorio MB. Quality improvement education in residency training: a review. *Am J Phys Med Rehabil*. 2018;97(9):673-678. https://doi.org/10.1097/PHM.0000000000000947
17. Vinas EK, White AB, Rogers RG, Ridgeway JJ, Young AE. Developing best clinical practices through outcomes improvement: an ongoing quality improvement curriculum for faculty and residents. *MedEdPORTAL*. 2018;14:10676. https://doi.org/10.15766/mep_2374-8265.10676
18. Reardon CL, Hafer R, Langheim FJP, et al. A quality improvement curriculum for psychiatry residents. *MedEdPORTAL*. 2020;16:10870. https://doi.org/10.15766/mep_2374-8265.10870
19. Worsham C, Swamy L, Gilad A, Abbott J. Quality improvement virtual practicum: the QI simulator. *MedEdPORTAL*. 2018;14:10670. https://doi.org/10.15766/mep_2374-8265.10670
20. Greenlaw C, Jacob S, Cheston CC. Pediatric quality improvement (QI) virtual practicum: adapting a QI simulator. *MedEdPORTAL*. 2020;16:10929. https://doi.org/10.15766/mep_2374-8265.10929
21. Moffatt-Bruce SD, Lee ME, Kneuertz PJ. Quality improvement in cardiothoracic surgery residency: training in the culture of change. *J Thorac Cardiovasc Surg*. 2020;160(5):1255-1260. https://doi.org/10.1016/j.jtcvs.2020.03.161

Copyright © 2021 Kristobak and Snider. This is an open-access publication distributed under the terms of the Creative Commons Attribution license.

22. Mrdutt MM, Isbell CL, Regner JL, et al. NSQIP-based quality improvement curriculum for surgical residents. *J Am Coll Surg*. 2017;224(5):868-874. https://doi.org/10.1016/j.jamcollsurg.2017.02.003

23. Chilkoti G, Mohta M, Wadhwa R, Saxena AK. Problem-based learning research in anesthesia teaching: current status and future perspective. *Anesthesiol Res Pract*. 2014;2014:263948. https://doi.org/10.1155/2014/263948

24. Carrero E, Gomar C, Penzo W, Rull M. Comparison between lecture-based approach and case/problem-based learning discussion for teaching pre-anaesthetic assessment. *Eur J Anaesthesiol*. 2007;24(12):1008-1015. https://doi.org/10.1017/S0265021506002304

25. Barrows HS. A taxonomy of problem-based learning methods. *Med Educ*. 1986;20(6):481-486. https://doi.org/10.1111/j.1365-2923.1986.tb01386.x

26. Liu PL, Liu LMP. A practical guide to implementing problem-based learning in anesthesia. *Curr Anaesth Crit Care*. 1997;8(4): 146-151. https://doi.org/10.1016/S0953-7112(97)80027-0

27. Davis WK, Oh MS, Anderson RM, Gruppen L, Nairn R. Influence of a highly focused case on the effect of small-group facilitators' content expertise on students' learning and satisfaction. *Acad Med*. 1994;69(8):663-669. https://doi.org/10.1097/00001888-199408000-00016

28. Bonnes SL, Ratelle JT, Halvorsen AJ, et al. Flipping the quality improvement classroom in residency education. *Acad Med*. 2017;92(1):101-107. https://doi.org/10.1097/ACM.0000000000001412

29. Silver SA, Harel Z, McQuillan R, et al. How to begin a quality improvement project. *Clin J Am Soc Nephrol*. 2016;11(5):893-900. https://doi.org/10.2215/CJN.11491015

30. Bassuk JA, Washington IM. The A3 Problem Solving Report: a 10-step scientific method to execute performance improvements in an academic research vivarium. *PLoS One*. 2013;8(10): e76833. https://doi.org/10.1371/journal.pone.0076833

31. Bowen DJ, Kreuter M, Spring B, et al. How we design feasibility studies. *Am J Prev Med*. 2009;36(5):452-457. https://doi.org/10.1016/j.amepre.2009.02.002

Received: April 14, 2021
Accepted: August 31, 2021
Published: November 29, 2021

Copyright © 2021 Kristobak and Snider. This is an open-access publication distributed under the terms of the Creative Commons Attribution license.
